# Thyroid hormone-induced expression of Foxl1 in subepithelial fibroblasts correlates with adult stem cell development during *Xenopus* intestinal remodeling

**DOI:** 10.1038/s41598-020-77817-1

**Published:** 2020-11-26

**Authors:** Takashi Hasebe, Kenta Fujimoto, Atsuko Ishizuya-Oka

**Affiliations:** Department of Biology, Nippon Medical School, 1-7-1 Kyonan-cho, Musashino, Tokyo 180-0023 Japan

**Keywords:** Developmental biology, Stem-cell niche, Stem cells, Stem cells, Adult stem cells, Intestinal stem cells, Stem-cell niche

## Abstract

In the *Xenopus laevis* intestine during metamorphosis, stem cells appear and generate the adult epithelium analogous to the mammalian one. We have previously shown that connective tissue cells surrounding the epithelium are essential for the stem cell development. To clarify whether such cells correspond to mammalian Foxl1-expressing mesenchymal cells, which have recently been shown to be a critical component of intestinal stem cell niche, we here examined the expression profile of Foxl1 in the *X. laevis* intestine by using RT-PCR and immunohistochemistry. Foxl1 expression was transiently upregulated only in connective tissue cells during the early period of metamorphic climax and was the highest just beneath the proliferating stem/progenitor cells. In addition, electron microscopic analysis showed that these subepithelial cells are ultrastructurally identified as telocytes like the mammalian Foxl1-expressing cells. Furthermore, we experimentally showed that Foxl1 expression is indirectly upregulated by thyroid hormone (TH) through Shh signaling and that TH organ-autonomously induces the Foxl1-expressing cells concomitantly with appearance of the stem cells in the tadpole intestine in vitro. The present results suggest that intestinal niche cells expressing Foxl1 are evolutionally conserved among terrestrial vertebrates and can be induced by TH/Shh signaling during amphibian metamorphosis for stem cell development.

## Introduction

In the mammalian intestine, the epithelium is rapidly renewed from stem cells localized in the crypt throughout the adulthood. The microenvironment around the stem cells called as the niche has been well documented to play important roles in maintaining the epithelial cell-renewal^1–4^ and is thus considered to be important for regenerative and cancer therapies. Although the molecular bases of the intestinal stem cell niche are still poorly understood, recent transgenic studies have demonstrated that a subset of subepithelial mesenchymal cells marked by the expression of the winged-helix transcription factor forkhead box l1 (Foxl1) is the critical niche component for maintaining the epithelial cell-renewal^5,6^. These Foxl1-expressing mesenchymal cells often coexpress Gli1^7^ and are morphologically termed “telocytes”^6,8^ which possess the long cell processes called as telopodes^9,10^. However, it still remains unclear how the Foxl1-expressing cells are differentiated and contribute to the formation of intestinal stem cell niche during mammalian postembryonic development in utero.

During amphibian metamorphosis, the digestive tract undergoes extensive remodeling from the larval to adult form. In the *Xenopus laevis* small intestine at Nieuwkoop and Faber (NF)^11^ stage 60 (early period of metamorphic climax), most of the larval epithelial cells begin to undergo apoptosis^12^, while a small number of them dedifferentiate into stem cells analogous to the mammalian ones that express intestinal stem cell markers such as the orphan leucine-rich repeat-containing G-protein-coupled receptor 5 (LGR5)^13^, Musashi-1 (Msi1)^14^, and c-Myc^15^. Histologically, the stem cells become detectable as small islets (primordia of the adult epithelium) between the degenerating larval epithelium and the connective tissue. The adult epithelial primordia actively proliferate during stages 60–62 and then, as multiple intestinal folds form, become differentiated into the simple columnar adult epithelium, where the stem cells are localized in the trough of intestinal folds^16^ similar to the mammalian ones localized in the crypt^17^. Since this intestinal remodeling can be easily induced by thyroid hormone (TH) both in vivo and in vitro^18^, the amphibian metamorphosis provides us an excellent opportunity to study molecular mechanisms underlying development of the stem cells. Previously, by using the *X. laevis* tadpole intestine, we experimentally demonstrated that connective tissue cells surrounding the epithelium are required for the adult stem cell development in the presence of TH^19^. In addition, our previous in situ hybridization (ISH) analyses of TH response genes indicated that the connective tissue cells close to the adult epithelial primordia highly express several signaling genes such as bone morphogenetic protein (BMP) 4^20^, patched 1 (Ptc1), Gli1^21^ and CD44^22^. These results strongly suggest that the connective tissue cells surrounding the adult epithelial primordia play important roles in the stem cell development as a niche component. However, it is not yet known whether these cells express Foxl1 just like the mammalian subepithelial Foxl1-expressing cells, and if so, what signals upregulate Foxl1 expression in the amphibian remodeling intestine. To address this issue, we here examined the expression profile of Foxl1 in the *X. laevis* intestine during metamorphosis. Our results indicate that Foxl1 is specifically expressed in the connective tissue cells surrounding the adult epithelial primordia and that its expression is indirectly upregulated by TH through sonic hedgehog (Shh) signaling. In addition, the amphibian Foxl1-expressing cells share common molecular and morphological characteristics with the mammalian ones, suggesting their evolutionally conserved roles as the intestinal stem cell niche.

## Results

### Upregulation of Foxl1 in the intestine during natural and TH-induced metamorphosis

**Figure 1:**
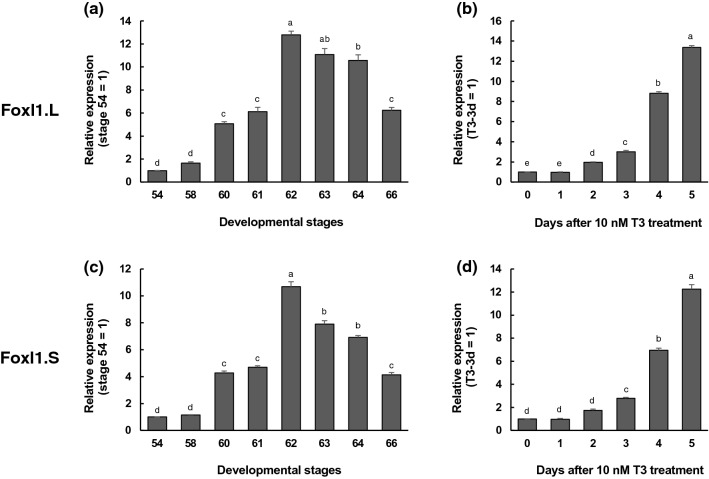
Expression profiles of Foxl1 homeolog mRNAs in the small intestine during natural and T3-induced metamorphosis. Total RNA was prepared from the intestine of *X. laevis* tadpoles at indicated developmental stages (**a**,**c**) or Nieuwkoop and Faber (NF) stage 54-tadpoles after 10 nM T3 treatment (**b**,**d**) and was analyzed by qRT-PCR. Levels of Foxl1.L (**a**,**b**) and Foxl1.S mRNAs (**c**,**d**) are shown relative to those of ribosomal protein L8 (rpL8.S) mRNA, with the values at stage 54 or T3 for 0 day set to 1^41–43^. Error bars represent the SEM (n = 4). The values were analyzed by ANOVA followed by Scheffe’s post hoc test. Different letters indicate significant differences among the indicated stages or days after T3 treatment (P < 0.05).

To study the temporal gene regulation of Foxl1 during intestinal remodeling, we first carried out real-time RT-PCR using total RNA extracted from the *X. laevis* small intestine at various metamorphic stages (Fig. 1). Since there are two homeologs of Foxl1 gene (Foxl1.L and Foxl1.S) in the allotetraploid genome of *X. laevis*^23^, we analyzed their expression levels by using the specific primers for each homeolog. The expression of Foxl1.L mRNA was upregulated at stage 60 (early period of metamorphic climax) and reached its peak at stage 62, when the adult epithelial cells are actively proliferating^12^. Thereafter, its expression decreased toward stage 66 (end of metamorphosis) (Fig. 1a). The expression prolife of Foxl1.S mRNA was similar to that of Foxl1.L mRNA (Fig. 1c).

Because TH is known to induce precocious intestinal remodeling, if these Foxl1 homeologs are involved in this process, their expression profiles should be reproduced during metamorphosis induced by exogenously-supplied TH. Thus, we analyzed their expression in the intestine of premetamorphic tadpoles at stage 54 treated with 10 nM T3 for 1 to 5 days. The expression of Foxl1.L mRNA was significantly upregulated after 2 days of T3 treatment and continued to rise even after 5 days of treatment (Fig. 1b). Again, similar results were obtained for Foxl1.S mRNA, except that its expression was significantly upregulated after 3 days of treatment (Fig. 1d).

These results suggest that both Foxl1 homeologs may play some roles in the intestinal remodeling and that their expression is upregulated by T3 in a similar manner. In addition, since the upregulation of gene expression of Foxl1 homeologs was relatively later than that of the direct TH response genes such as Shh^21,24^ and was not drastic until 3 days of treatment (up to threefold), it seems likely that their expression is indirectly regulated by T3.

### TH indirectly induces Foxl1 expression through upregulation of Shh

**Figure 2:**
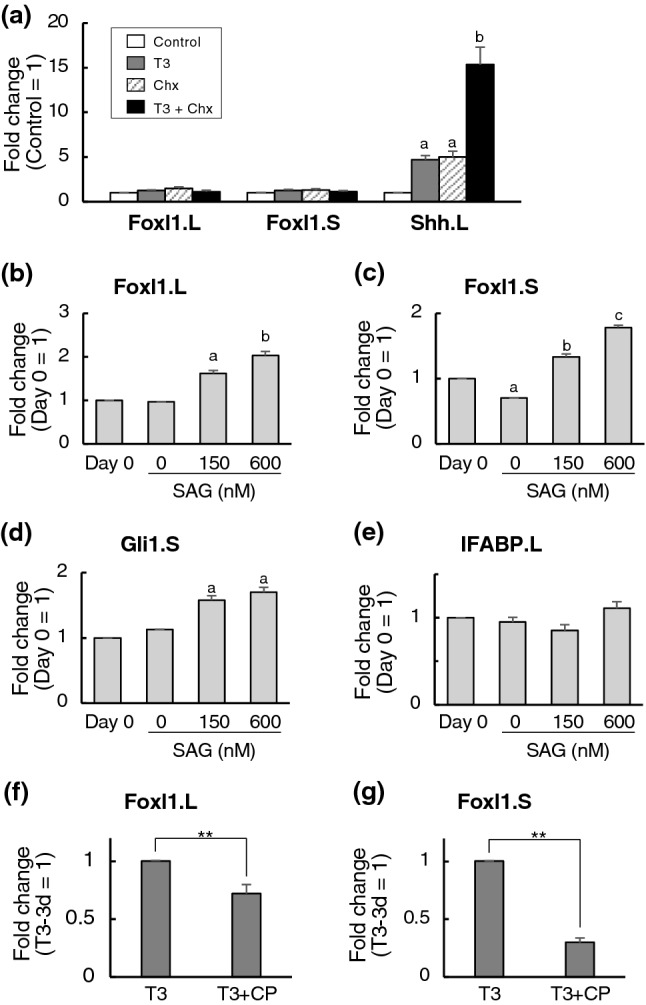
T3 indirectly induces the mRNA expression of Foxl1 homeologs through Shh signaling. (**a**) Total RNA was isolated from the intestine of premetamorphic stage 54-tadpoles treated with DMSO (Control, white bars), 50 nM T3+DMSO (T3, gray bars), cycloheximide and anisomycin (Chx, shaded bars) and 50 nM T3+Chx (T3+Chx, black bars) for 6 h following the pretreatment with DMSO or Chx for 1 h. mRNA levels of indicated genes were examined by qRT-PCR and shown relative to those of rpL8.S mRNA, with the value of Control set to 1. Error bars represent the SEM (n = 4). The values were analyzed by ANOVA followed by Scheffe’s post hoc test. Different letters indicate significant differences among the indicated treatment (P < 0.05). When both Control vs T3 and Chx vs T3+Chx were statistically significant, the gene was determined as the direct TH response gene. (**b**–**e**) Premetamorphic tadpoles at stage 54 were pretreated with DMSO for 4 days. Then, total RNA was isolated from the intestine at 0 day or 1 day after treatment with 0, 150 or 600 nM SAG. mRNA levels of indicated genes were examined by qRT-PCR and shown relative to those of rpL8.S mRNA, with the value at day 0 set to 1. Error bars represent the SEM (n = 6 for Foxl1.L, n = 5 for Foxl1.S, n = 3 for Gli1.S and IFABP.L). The values were analyzed by ANOVA followed by Scheffe’s post hoc test. Different letters indicate significant differences among the indicated treatment (P < 0.05). (**f**,**g**) Premetamorphic tadpoles at stage 54 were treated with 10 nM T3 in the presence of 2.5 mM cyclopamine (CP) or ethanol vehicle for 3 days. Then, total RNA was isolated from the intestine. mRNA levels of indicated genes were examined by qRT-PCR and shown relative to those of rpL8.S mRNA, with the value of T3 plus ethanol vehicle for 3 days set to 1. Error bars represent the SEM (n = 3). The values were analyzed by Student’s *t*-test. Asterisks indicate that the mRNA levels are significantly different. **P < 0.01.

The regulation of direct TH target genes is mediated by the thyroid hormone receptor (TR) immediately upon T3 addition and is thus independent of new protein synthesis. To determine whether Foxl1 homeologs are direct transcriptional targets of TH or not, protein synthesis inhibitors, cycloheximide and anisomycin (Chx) were added to the rearing water of premetamorphic tadpoles at stage 54 for 1 h before and throughout 50 nM T3 treatment for 6 h to block protein synthesis^25^ (Fig. 2a). The mRNA expression of the positive control, Shh, a known TH direct response gene^24^, was significantly upregulated by T3 even in the presence of Chx. In contrast, the expression of neither Foxl1.L nor Foxl1.S mRNA was significantly upregulated by T3 in the presence of Chx, indicating that their expression is not directly regulated by TH.

In an attempt to find out potential transcriptional regulators of Foxl1 genes, we focused on Shh signaling, since the cells expressing Foxl1 often coexpress Gli1^7^, a transcriptional mediator of Shh signaling^26,27^ and Shh is the direct target of T3^21,24^. By using the web tool (http://jaspar.genereg.net), we found several putative Gli-binding sites in both Foxl1.L and Foxl1.S genes (not shown) in addition to those reported by Madison et al.^28^. Then, we pretreated premetamorphic tadpoles at stage 54, when the expression level of Shh in the intestine is known to be very low^24^, with DMSO for 4 days followed by treatment with 0, 150 or 600 nM Smoothened agonist (SAG) for 1 day to activate Shh signaling^29^ independently of T3. The expression of both Foxl1.L and Foxl1.S mRNAs was significantly upregulated by SAG in a dose-dependent manner (Fig. 2b,c). These results were comparable to that for Gli1, a known target of Shh signaling^30^ (Fig. 2d). In contrast, the mRNA expression of intestinal fatty acid-binding protein (IFABP), which is specifically expressed in differentiated absorptive epithelial cells independently of Shh signaling^31^, was not affected by SAG treatment (Fig. 2e). In addition, to inhibit Shh signaling, premetamorphic tadpoles at stage 54 were treated with 2.5 mM cyclopamine (CP), an inhibitor of Shh signaling^32^, in the presence of T3 for 3 days. TH-upregulated expression of both Foxl1 mRNAs was significantly suppressed by CP (Fig. 2f,g). Taken together, our results suggest that TH-activated Shh signaling upregulates the expression of Foxl1 genes through the action of Gli1.

### Foxl1 expression profile correlates with intestinal stem cell development

**Figure 3:**
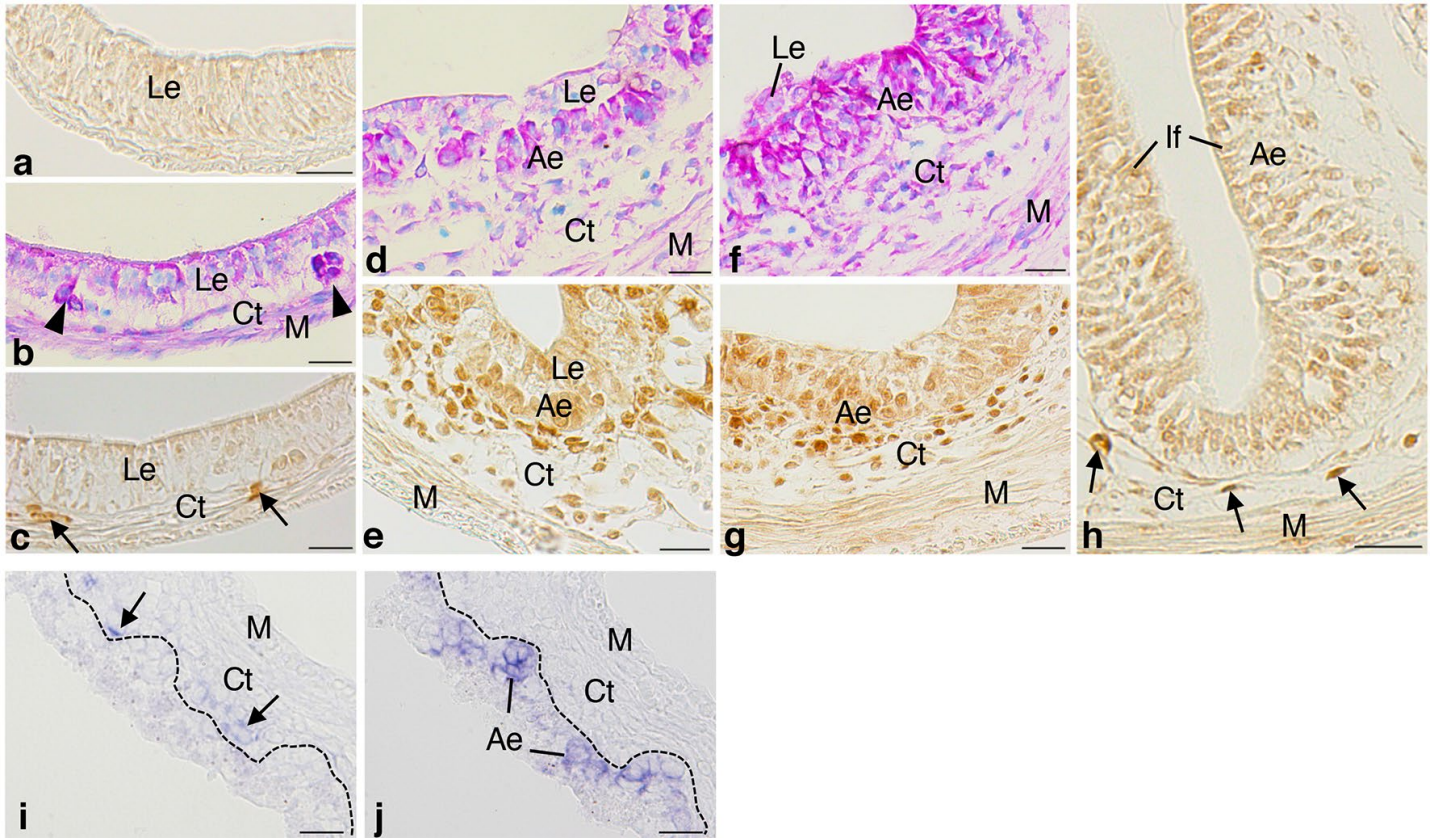
Spatio-temporal expression of Foxl1 in the *X. laevis* intestine during metamorphosis. Cross sections were immunostained with anti-Foxl1 antibody (**a**,**c**,**e**,**g**,**h**) and stained with methyl green-pyronin (**b**,**d**,**f**), or hybridized with antisense Foxl1 (**i**) or c-Myc probes (**j**). (**a**) Stage 57. No cell is positive for Foxl1. (**b**,**c**) Stage 60. Adult stem/progenitor cells appear as islets strongly stained red with pyronin ((**b**), arrowheads) between the larval epithelium (Le) and the connective tissue (Ct). Some connective tissue cells become positive for Foxl1 ((**c**), arrows). (**d**,**e**) Stage 61. Both primordia of the adult epithelium (Ae) and the connective tissue increase in cell number (**d**). Connective tissue cells positive for Foxl1 become numerous close to the adult epithelial primordia (**e**). (**f**,**g**) Stage 62. The adult epithelium mostly replaces the larval one (**f**). Most of the connective tissue cells surrounding the adult epithelium are positive for Foxl1 (**g**). (**h**) Stage 66 (end of metamorphosis). Connective tissue cells positive for Foxl1 (arrows) are small in number and tend to be localized in the trough of newly-formed intestinal folds (If). (**i**,**j**) Stage 62. Foxl1 mRNA (**i**, arrows) is detected as dark blue deposits in the connective tissue cells close to adult epithelial primordia expressing c-Myc (**j**). The dashed-lines indicate the boundary of the epithelium and connective tissue. M; muscles. Bars, 20 μm.

We next immunohistochemically examined spatio-temporal correlations between Foxl1 expression and adult epithelial development in the *X. laevis* small intestine. Before metamorphic climax, the tadpole small intestine mainly consists of the simple columnar larval epithelium, the immature connective tissue, and thin layers of muscles. Every tissue was negative for Foxl1 throughout the larval period until stage 59 (Fig. 3a), in agreement with very weak Foxl1 expression detected by RT-PCR during this period.

At stage 60, when adult stem cells become detectable as small islets stained red with pyronin (primordia of the adult epithelium) between the larval epithelium and the developing connective tissue in the entire small intestine (Fig. 3b)^12^, cells positive for Foxl1 became detectable only in the connective tissue underneath the epithelium (Fig. 3c). At stage 61 when both the adult epithelial primordia and the connective tissue rapidly increase in cell number (Fig. 3d), the connective tissue cells positive for Foxl1 increased in number close to the adult epithelial primordia (Fig. 3e) and then, at stage 62 when the adult epithelium mostly replaces the larval one (Fig. 3f), most of the connective tissue cells surrounding the adult epithelium were positive for Foxl1 (Fig. 3g). Thereafter, as multiple intestinal folds form towards stage 66, the connective tissue cells positive for Foxl1 rapidly decreased in number and became mostly localized in the trough of intestinal folds (Fig. 3h), where the stem cells are localized^13^. In agreement with the immunohistochemical analysis described above, ISH analysis showed that Foxl1 mRNA was specifically expressed in the connective tissue cells (Fig. 3i) underneath the adult epithelial primordia expressing c-Myc, one of the intestinal stem cell markers^15^, at stage 62 (Fig. 3j).

**Figure 4:**
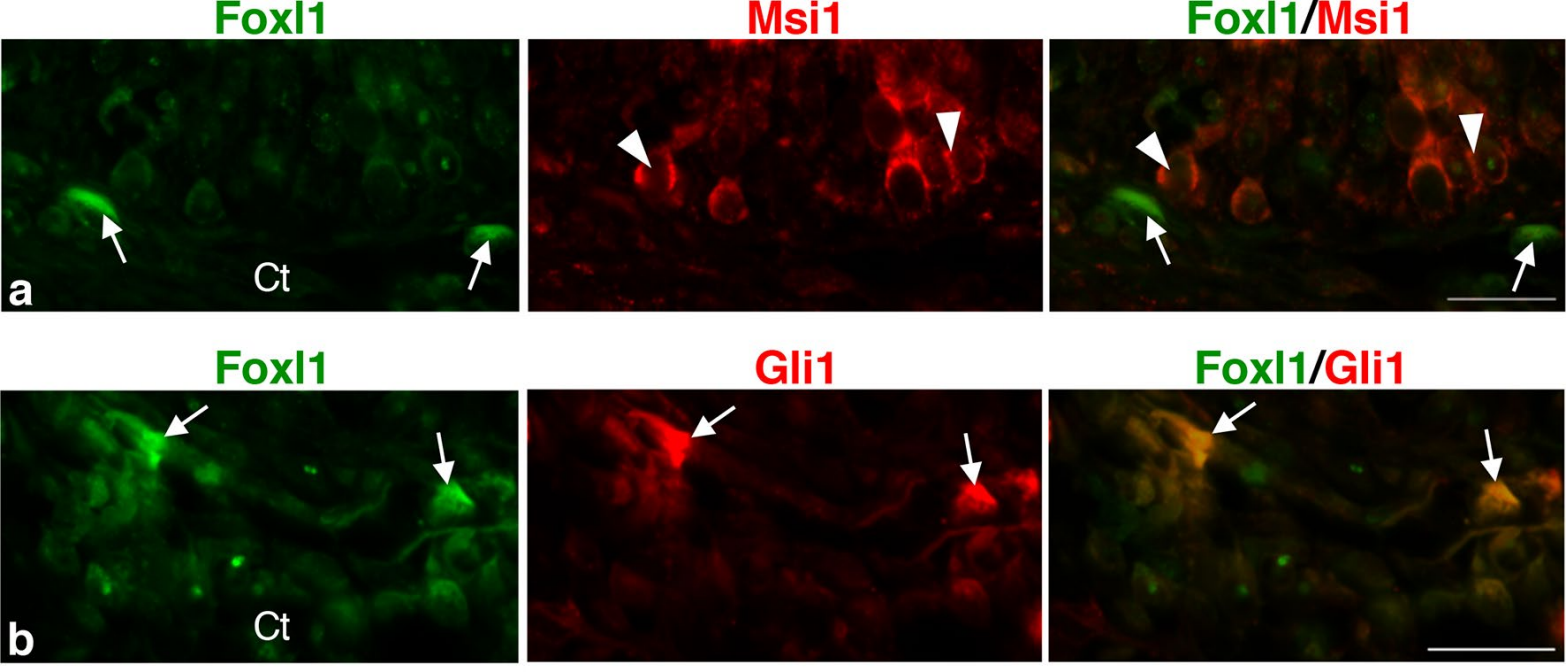
Correlation between Foxl1 expression and the expression of Msi1, an adult stem cell marker, and that of Gli1 in the *X. laevis* intestine at stage 61. Cells positive for Foxl1 (**a**,**b**), arrows) are localized in the connective tissue (Ct) close to primordia of the adult epithelium expressing Msi1 ((**a**), arrowheads). Most of them are also positive for Gli1 ((**b**), arrows). Bars, 20 μm.

To investigate the spatio-temporal correlation between the cells positive for Foxl1 and the adult epithelial primordia more directly, double-immunofluorescence labeling was performed in the *X. laevis* intestine at stage 61. Cells positive for Foxl1 were localized in the connective tissue close to the adult epithelial primordia positive for Msi1 (Fig. 4a), one of the stem cell markers common to the mammalian and amphibian intestines^14,33^. In addition, most of the cells positive for Foxl1 were also positive for Gli1 (Fig. 4b), whose expression agrees well with its mRNA expression shown by our previous ISH study^21^.

### Ultrastructure of subepithelial fibroblasts surrounding adult epithelial primordia

**Figure 5:**
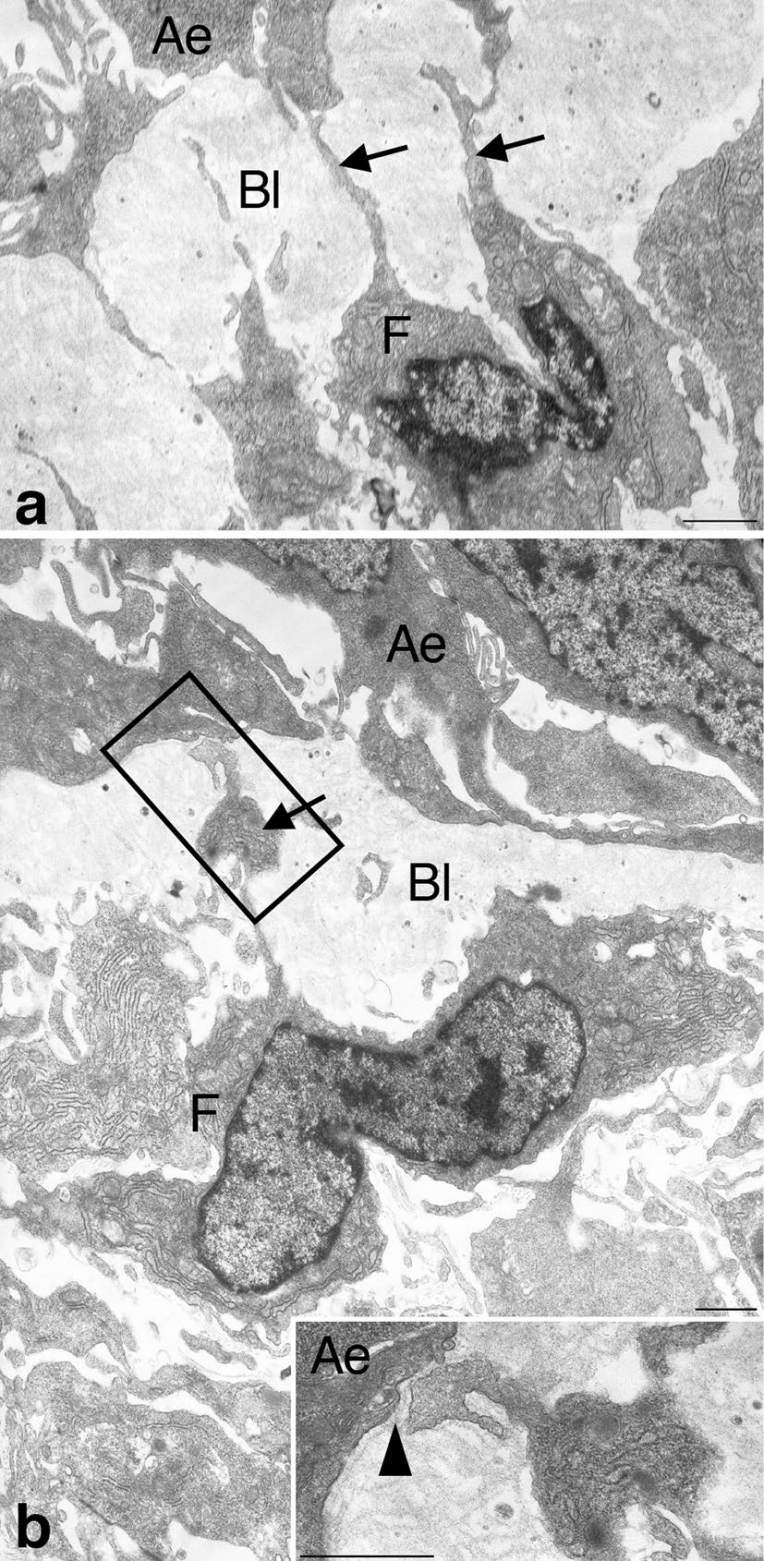
Epithelial-connective tissue interfaces in the *X. laevis* intestine during stages 61–62. Subepithelial fibroblasts (F) often possess long and thin processes ((**a**), arrows) and make contacts with stem/progenitor cells of the adult epithelium (Ae) through the modified basal lamina (Bl). Some of the processes are occasionally dilated ((**b**), arrow). Inset: Higher magnification of the boxed area in (**b**). The process makes contact with the adult epithelial cell (arrowhead). Bars, 1 μm.

To obtain more information about the morphological characteristics of the Foxl1-expressing cells, we observed by electron microscopy the connective tissue of the *X. laevis* intestine during stages 61–62, when almost all of the connective tissue cells just beneath the adult epithelial primordia express Foxl1. During this period, the subepithelial connective tissue cells were mainly irregular-shaped fibroblasts. Characteristically, they possessed many long and thin processes whose diameter was mostly less than 200 nm (Fig. 5a) In addition, some of the processes had a moniliform aspect with dilations where the cell organelles often existed (Fig. 5b). Through these long processes, the subepithelial cells often made contact with the adult epithelial primordia (Fig. 5a,b). These ultrastructural characteristics coincide well with those of telocytes possessing long processes termed “telopodes”^34^, as well as the Foxl1-expressing cells in the mammalian adult intestine^35^.

### TH organ-autonomously upregulates Foxl1 expression in vitro

**Figure 6:**
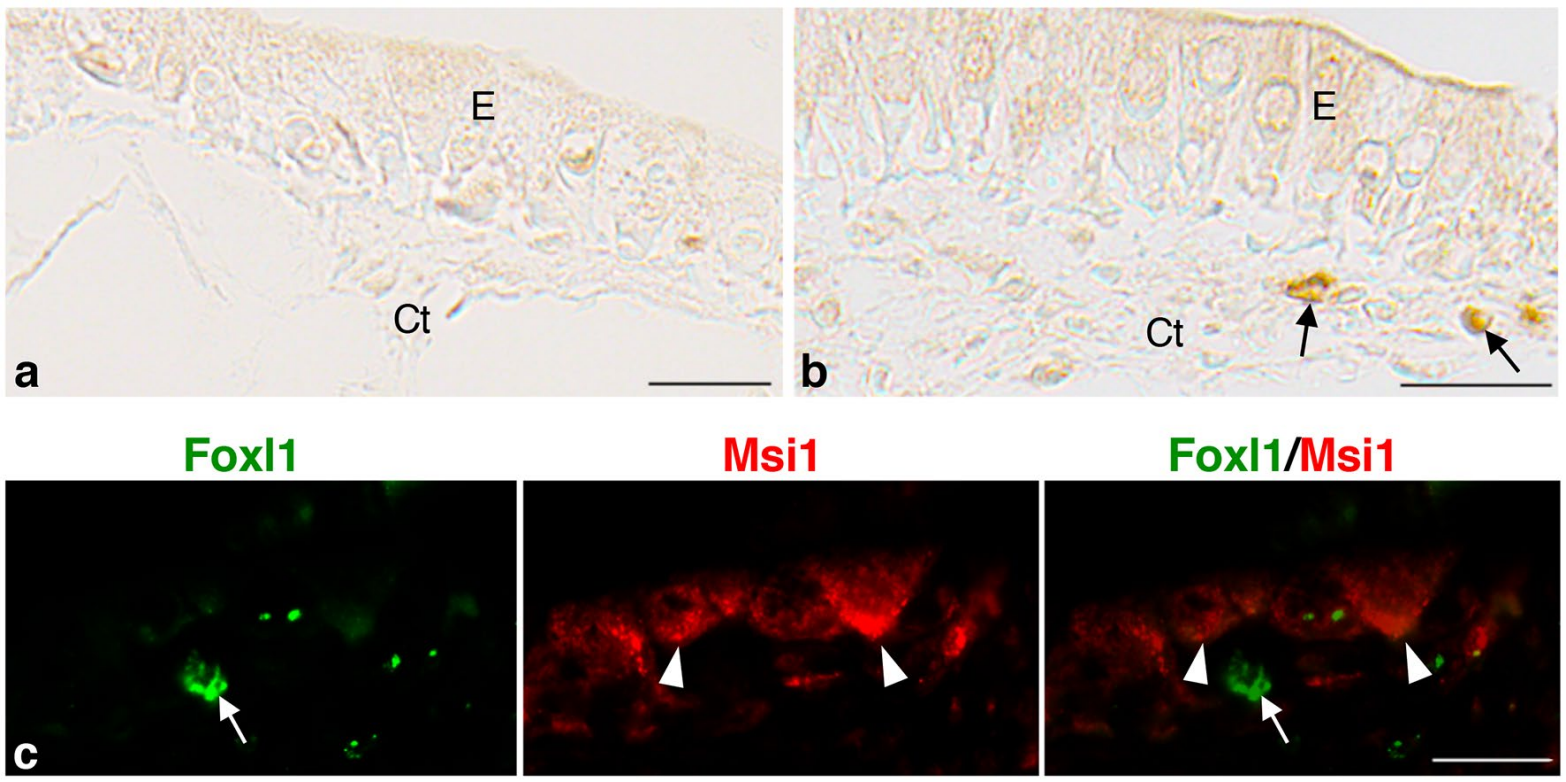
T3-induced expression of Foxl1 in the connective tissue of the *X. laevis* tadpole intestine cultured for 5 days in vitro. Cross sections were immunostained with anti-Foxl1 antibody (**a**,**b**) and double-immunostained with anti-Foxl1 and anti-Msi1 antibodies (**c**). (**a**) Control intestine cultured in the absence of T3. The epithelium (E) remains simple columnar. No cell is positive for Foxl1 in any tissue. (**b**,**c**) Intestines cultured in the presence of T3. Cells positive for Foxl1 are detectable in the connective tissue (Ct) but not in the epithelium (**b**), arrows). These cells ((**c**), arrow) are localized near adult epithelial primordia expressing Msi1 ((**c**), arrowheads). Bars, 20 μm.

Finally, to clarify whether the Foxl1-expressing cells are organ-autonomously induced by TH in vitro, we cultured the small intestine isolated from *X. laevis* stage 57-tadpoles in the presence or absence of T3. In its absence, the larval epithelium remained simple columnar, and every tissue was negative for Foxl1 throughout the cultivation (Fig. 6a). By contrast, in the presence of T3, although the intensity of Foxl1 immunoreactivity was lower than in vivo, cells positive for Foxl1 became detectable around culture day 5 (Fig. 6b) when adult epithelial primordia appear in vitro (Fig. 6c)^14^. They were localized in the connective tissue close to the adult epithelial primordia expressing Msi1 (Fig. 6c) just like those during natural metamorphosis. These results indicate that the Foxl1-expressing cells originate from the tadpole intestine itself before metamorphic climax.

## Discussion

In the present study, we have shown by using RT-PCR that the expression of Foxl1 gene homeologs, both Foxl1.L and Foxl1.S, temporally correlates well with stem cell development in the *X. laevis* intestine during metamorphosis. In addition, although it is difficult to distinguish the cells expressing each homeolog of Foxl1 gene by immunohistochemistry or ISH at present, we found that Foxl1 is specifically expressed in the connective tissue cells, which often coexpress Gli1 and are morphologically identified as telocytes just like the mammalian Foxl1-expressing cells essential for maintaining the epithelial cell-renewal^8^. These findings strongly suggest that Foxl1-expressing cells common to the mammalian ones appear in the amphibian intestine when the stem cells develop during metamorphosis. Therefore, it should be an interesting future topic to clarify the role of Foxl1 in stem cell development of the metamorphosing intestine.

In the *X. laevis* intestine, we have previously shown that TH directly upregulates the expression of Shh only in the epithelium and that Shh expression reaches its peak in the adult epithelial primordia that actively proliferate^36,37^. By contrast, a Shh receptor, Ptc1, and its downstream transcription factors Glis are specifically expressed in the connective tissue cells^21^. In the present study, we demonstrated that TH indirectly upregulates the expression of Foxl1, both Foxl1.L and Foxl1.S homeologs, by activating Shh signaling and that the expression of Foxl1 at least partly overlaps with that of Gli1, whose expression is transiently up-regulated in the connective tissue during metamorphic climax and reaches its maximum level at stage 62^21^ similar to the Foxl1 expression. Taken together with the fact that Foxl1 is a target of Gli proteins, we here propose that, in the amphibian intestine, TH induces the adult epithelial primordia to express Shh, which in turn acts on the nearby connective tissue cells to express Foxl1 via Gli1 in a paracrine manner. In the developing mammalian intestine, the expression of Foxl1 has been also shown to be up-regulated by Shh through Gli binding sites^28^, but it still remains unknown whether TH regulates the Foxl1 expression. Although the origin of the Foxl1-expressing cells remains unclear, our culture study indicated that they are derived from the tadpole intestine itself before metamorphic climax. Since the Shh receptor Ptc1 is expressed only in the connective tissue cells^21^, it can be concluded that the Foxl1-expressing cells originate from some cells residing in the connective tissue of the tadpole intestine. Thus, our next step should be directed to clarify what type of larval connective tissue cells can be induced by Shh signaling to differentiate into telocytes that express Foxl1 by making use of transgenic frogs in which the Foxl1-expressing cells are genetically labeled.

Our previous studies have elucidated several signals important for the stem cell development in the *X. laevis* intestine, such as Shh, BMP4, and Wnts^16^. It should be noted that the expression profile of Foxl1 shown by the present study spatio-temporally coincides well with that of BMP4, which is highly expressed in almost all of the connective tissue cells surrounding the adult epithelial primordia and is also upregulated by TH/Shh signaling^38^. Thus, it seems highly likely that Shh-upregulated expression of BMP4 is mediated by Foxl1, as reported in the mammalian intestine where BMP4 is a downstream target of Foxl1^28,39^. Furthermore, in the mammalian intestine, the Foxl1-expressing cells have recently been shown to be a source of Wnts essential for maintaining the epithelial cell-renewal^5,6^. So far, in the *X. laevis* intestine, Wnt2b and Wnt5a have been identified as TH response genes^40^. In addition, we have previously shown that both canonical and non-canonical Wnt signaling pathways are activated in the stem cells during development^22,41,42^. Thus, to clarify which Wnt genes are the targets of Foxl1 and contribute to the formation of the stem cell niche, the expression and functional analyses of Wnt genes should be further performed in the *X. laevis* intestine.

In conclusion, we have shown here that subepithelial Foxl1-expressing cells, which shares common molecular and morphological characteristics with mammalian ones, appear by the inductive action of TH/Shh signaling in the amphibian intestine during metamorphosis. To understand the evolutionally conserved molecular mechanisms underlying the stem cell niche formation, it is a challenging future issue to clarify how and what kinds of signals are exchanged between the Foxl1-expressing cells and the nearby stem cells during postembryonic development by making use of gene knockout technologies in the present animal model.

## Materials and methods

### Animals and treatment

Tadpoles of the South African clawed frogs (*Xenopus laevis*) at Nieuwkoop and Faber (NF) stages^11^ from 54 (premetamorphosis) to 66 (end of metamorphosis) were reared in the laboratory and used throughout the experiments. Some tadpoles at stage 54 were treated with 10 nM 3, 3′, 5-triiodo-L-thyronine (T3) (Sigma-Aldrich, St. Louis¸ MO, USA), whose stock solution was prepared at 50 μM in 10 mM NaOH, for 1 to 5 days. To inhibit protein synthesis, cycloheximide and anisomycin (Chx) dissolved in dimethyl sulfoxide (DMSO) were added to the rearing water of premetamorphic stage 54-tadpoles at 20 and 25 mg/l, respectively (final concentration of DMSO was 0.1%). It has been reported that Chx treatment at this concentration for 1 h inhibits more than 99% of protein synthesis in the stage-54 tadpoles^25^. 0.1% DMSO was used for the control. After 1 h of Chx treatment, T3 was added at 50 nM, and the tadpoles were additionally treated for 6 h. To activate Shh signaling, Smoothened agonist (SAG, Enzo Life Sciences, Farmingdale, NY, USA) dissolved in DMSO at 3 or 0.75 mM was used^29^. Premetamorphic tadpoles at stage 54, when the expression level of Shh is very low in the intestine^24,36^, were pretreated with 0.02% DMSO for 4 days. Then, the tadpoles were treated with 0, 150 or 600 nM SAG for 1 day (final concentration of DMSO was 0.02%). To inhibit Shh signaling, cyclopamine (CP, Fujifilm Wako pure chemical, Osaka, Japan) dissolved in 100% ethanol at 5 mM was used^32^. Premetamorphic tadpoles at stage 54 were treated with 10 nM T3 in the presence of 2.5 mM CP or 0.05% ethanol vehicle for 3 days. At least three tadpoles were analyzed for each stage or day of T3, SAG or CP treatment. All experiments using the *X. laevis* animals were approved by the Animal Use and Care Committee of Nippon Medical School and were carried out in accordance with the relevant guidelines and regulations set by this committee.

### Real-time RT-PCR

Total RNA was extracted from the small intestine at the indicated developmental stages by using TRI Reagent (Molecular Research Center, Cincinnati, OH, USA) followed by DNase treatment with DNA-free (Ambion, Austin, TX, USA) to remove any DNA contamination. The integrity of RNA was checked based on 18S and 28S ribosomal RNAs by electrophoresis. Total RNA was mixed with GoTaq 1-Step RT-qPCR System (Promega, Madison, WI, USA), and then quantitative real-time RT-PCR was performed by using PikoReal 96 Real-Time PCR System (Thermo Fisher Scientific, Waltham, MA, USA) according to the manufacturer’s instructions. The primer pairs used are: 5′-TGCCGGGAGCCCAACTGATGC-3′ and 5′-TCGGACAGGAGTGGGACAGACAAG-3′ for Foxl1.L (GenBank: XM_018257951), 5′-CAGGAACTCTCTCCGGCTTC-3′ and 5′-CCCTTTCTGGCCCATACAGG-3′ for Foxl1.S (GenBank: XM_018259925), 5′-AGCGACTTCCTCATGTTCATC-3′ and 5′-GCCTTCAAGGTCATGGTCTTG-3′ for Shh.L (GenBank: NM_001088313)^36^, 5′-GCAGGTGAAGGAAATAATGGTCAG-3′ and 5′-TGTGCTGGTTTTGCCGAGTG-3′ for Gli1.S (GenBank: XM_018250072), 5′-CAGTGAAAGAATCCAGCACATTCC-3′ and 5′-TGTAGGAACCAGGCACCATTGAG-3′ for IFABP.L (GenBank: NM_001085877), 5′-GCCGTGGTGCTCCTCTTGCC-3′ and 5′-TGCCACAGTACACAAACTGTCCG-3′ for ribosomal protein L8 (rpL8.S; GenBank: NM_001086996). The level of specific mRNA was normalized against the level of rpL8.S mRNA^43,44^ for each sample. The samples were analyzed in duplicate at least 3 times. The specificity of the amplification was confirmed by the dissociation curve analysis. PCR without reverse transcription did not show any specific signal (not shown). The level of rpL8.S mRNA did not change throughout metamorphosis or after T3, SAG or CP treatment (not shown). The results were analyzed by ANOVA followed by Scheffe's post hoc test or Student's *t*-test.

### Immunohistochemistry (IHC)

Tubular fragments isolated from the anterior part of the small intestine just behind the bile duct junction and cultured intestinal explants were fixed with 95% ethanol at 4 °C for 4 h, embedded in paraffin, and cut at 5 μm. Some sections were immunostained with the rabbit anti-Foxl1 (1:100; LSBio, Seattle, WA, USA) antibody at room temperature for 1 h and then incubated with biotin-labeled anti-IgG and peroxidase-conjugated streptavidin (Nichirei, Tokyo, Japan) followed by 0.02% 3, 3′-diamino-benzidine-4HCl (DAB) and 0.006% H_2_O_2_. In addition, to distinguish conventionally adult stem/progenitor cells from the remaining larval cells during stages 60–62 when the larval-to-adult epithelial replacement occurs, other sections were stained with methyl green-pyronin (Muto, Tokyo, Japan) for 5 min^12^. The adult stem/progenitor cells were stained intensely red with pyronin because of their RNA-rich cytoplasm^45^, whereas the larval epithelial cells undergoing apoptosis were stained much weaker during this period. Furthermore, some other sections were double-immunostained at room temperature for 1 h with mixtures of the rabbit anti-Foxl1 (1:100) and the mouse anti-Musashi-1 (Msi1) (1:10; Abnova, Taipei, Taiwan) or anti-Gli1 antibodies (1:20; NovusBio, Centennial, CO, USA). They were then incubated with a mixture of Alexa Fluor 568-conjugated anti-rabbit IgG (1:500; Molecular Probes, Eugene, OR, USA) and Alexa Fluor 488-conjugated anti-mouse IgG antibodies (1:500; Molecular Probes) and analyzed by fluorescence microscopy. All the control sections showed only background levels of signals, if any (data not shown). According to the manufacturer's information, the human immunogen sequences used to raise the antibodies above are nearly identical to the *X. laevis* counterparts; anti-Foxl1 (96% to Foxl1.L, 100% to Foxl1.S), anti-Msi1 (92% to both Msi1.L and S), and anti-Gli1 antibodies (78% to both Gli1.L and S). At least three intestinal fragments were examined for each developmental stage, and six for each experimental time point after T3 treatment.

### In situ hybridization (ISH)

*X. laevis* genomic region containing Foxl1.L gene that consists of a single exon common to both Foxl1.L and Foxl1.S homeologs was cloned from the genomic DNA extracted from the intestine at stage 62 by PCR with primers (5′-GGGCAGATATTACTGACTTGATAGTAGGAGC-3′ and 5′-CCTGAGATAATAGGAGTGTATGGGAACAGGAC-3′). The amplified DNA fragment was used as a template for the nested PCR performed with primers (5′-AATATTGAATTCCTTCCACTACATCCTCACATTGCCCATGTATGTG-3′ and 5′-AATATTCCCGGGTTAAACAGCATTTGGAGTGTGACAGGTTTTCCAC-3′). The PCR product (ca. 5 kb) was double-digested with EcoRI and XmaI and inserted into pBSII-KS+ vector predigested with the same enzymes. Then, the DNA sequence was checked (pBSII-KS+_Foxl1.L(full)).

*X. laevis* c-Myc.S (stem cell-associated transcription factor)^15^ cDNA was isolated by PCR with primers (5′-GAGACCAATCGCATGGCAGG-3′ and 5′-GGAAGAGACAACCTTGATATAG-3′) using a cDNA prepared from the stage-62 intestine as a template followed by the nested PCR with primers (5′-AATATTACCGGTGCCGCCACCATGGCAGGAAAGATGCCTCTTAATG-3′ and 5′-TATTACTAGTCTACTTGTCGTCGTCATCCTTGTAGTCGACAAAGTTCCTCAGCTGTTGGAG-3′). The amplified DNA was double-digested with AgeI and SpeI and inserted into pT7Ts vector^46^ predigested with the same enzymes and sequenced (pT7Ts_c-Myc-FLAG). Nearly full-length of c-Myc.S coding region (ca. 1.2 kb) was digested out from this plasmid with BbvCI (followed by blunt-ending) and AgeI, and inserted into pBSII-KS+ vector predigested with HincII and XmaI (pBSII-KS+_c-Myc probe).

These plasmids were linearized to synthesize sense and antisense probes with T7 and T3 RNA polymerase, respectively, by using digoxigenin (DIG) RNA Labeling Mix (Roche Applied Science, Indianapolis, IN, USA). The probes were digested by alkaline treatment (40 mM NaHCO_3_, 60 mM Na_2_CO_3_) into ca. 200 base-long. Intestinal fragments were isolated from the anterior part of the small intestine just after the bile duct junction in tadpoles at stage 62 and fixed in MEMFA followed by cryosectioning. Tissue sections were prepared at 7 μm and subjected to ISH. ISH was performed by using the sense or antisense probe as previously described^47^. Sense probes did not show any specific signal (not shown). Photographs were taken by using a digital CCD color camera (WRAYCAM-ALASKA, Wraymer, Osaka, Japan) attached to an optical microscope (BX51, Olympus, Tokyo Japan).

### Transmission electron microscopy (TEM)

Small fragments isolated from the anterior part of the small intestine were fixed in 2.5% glutaraldehyde in 0.1 M cacodylate buffer (pH7.4) at 4 °C for 2 h and postfixed in 1% osmium tetroxide in the same buffer at 4 °C for 2 h. They were then stained en bloc with saturated uranyl acetate and embedded in epoxy resin. Transverse ultrathin sections were stained with 4% lead citrate for 5 min and were examined with a JOEL 200CX electron microscope.

### Organ culture

Tissue fragments were isolated from the anterior part of the small intestine just behind the bile duct junction in *X. laevis* tadpoles at stage 57 (before metamorphic climax) and were cultured for 5 days at 26 °C as we previously described^14,48^. Briefly, they were slit open lengthwise and placed on membrane filters (type HAWP; Millipore, Bedford, MA, USA) put on steel grids in culture dishes. The medium contains 60% Leibovitz’s L-15 medium (Gibco-BRL, Grand Island, NY, USA) supplemented with 100 IU/ml of penicillin, 100 μg/ml of streptomycin, and 10% charcoal-treated fetal bovine serum (Gibco-BRL). To induce metamorphic changes, 20 nM T3, 5.0 μg/ml insulin, and 0.5 μg/ml hydrocortisone (Sigma-Aldrich, Louis, MO, USA) were added to the medium. The culture medium was changed every other day.

### Western blot analysis

Using pBSII-KS+_Foxl1.L(full) as the template, Foxl1.L coding region fused with FLAG tag was amplified with primers (5′-AATATTACCGGTCCGCCACCATGGACTACAAGGATGACGACGACAAGAGCAGGATATACAGCAGCCCCCTGCC-3′ and 5′-AATATTGCTAGCCTCGAGCTATTGGGCAAAATGTGCATCTG-3′). The PCR product (ca. 1.2 kb) was digested with AgeI and NheI and inserted into pT7Ts vector predigested with AgeI and SpeI (pT7Ts_FLAG-Foxl1.L). This plasmid was linearized with BamHI to synthesize the capped mRNA with mMessage-mMachine T7 transcription kit (Thermo Fisher Scientific). The coding region of Msi1.L (ca. 1 kb) was digested out from nrp1-pSP36T^14^ by NcoI and SpeI and inserted into pT7Ts vector predigested with the same enzymes (pT7Ts_Msi1). The *X. laevis* proteins were produced by injecting capped mRNA into *X. laevis* embryos^46^ for Foxl1.L. The embryo extracts (input) and the extracts immunoprecipitated (FLAG-IP) by anti-FLAG agarose (Sigma-Aldrich) were prepared. The other proteins were produced by TNT T7 Quick Coupled Transcription/Translation System (Promega) with pT7Ts_FLAG-Foxl1.L and pT7Ts_Msi1 for Foxl1.L and Msi1.L, respectively, and by TNT SP6 (Promega) with pCS2-fGli1-myc^21^ for Gli1.S according to the manufacturer's instructions. To confirm that the antibodies specifically recognize the *X. laevis* proteins, the protein samples were subjected to Western blotting using anti-Foxl1 (1:250), anti-Msi1 (1:100) or anti-Gli1 (1:200). The specific signal was observed for each antibody (Fig. S1).

## Supplementary information

Supplementary Information.
