# Supplementary material for: Thyroid hormone-induced expression of Foxl1 in subepithelial fibroblasts correlates with adult stem cell development during Xenopus intestinal remodeling
Source: Sci Rep. 2020 Nov 26;10:20715. doi: 10.1038/s41598-020-77817-1 (PMC7693326; doi:10.1038/s41598-020-77817-1)
Supplement: Supplementary file 1 — Supplementary Information. [file 41598_2020_77817_MOESM1_ESM.docx]

**Supplementary Material**

**Thyroid hormone-induced expression of Foxl1 in subepithelial fibroblasts correlates with adult stem cell development during *Xenopus* intestinal remodeling**

Takashi Hasebe, Kenta Fujimoto & Atsuko Ishizuya-Oka*

Department of Biology, Nippon Medical School, Musashino, Tokyo 180-0023, Japan

*Correspondence to: Atsuko Ishizuya-Oka, Department of Biology, Nippon Medical School, 1-7-1 Kyonan-cho, Musashino, Tokyo 180-0023, Japan.

E-mail: [a-oka@nms.ac.jp](mailto:a-oka@nms.ac.jp), Tel: +81-422-34-3429, Fax: +81-422-34-1120


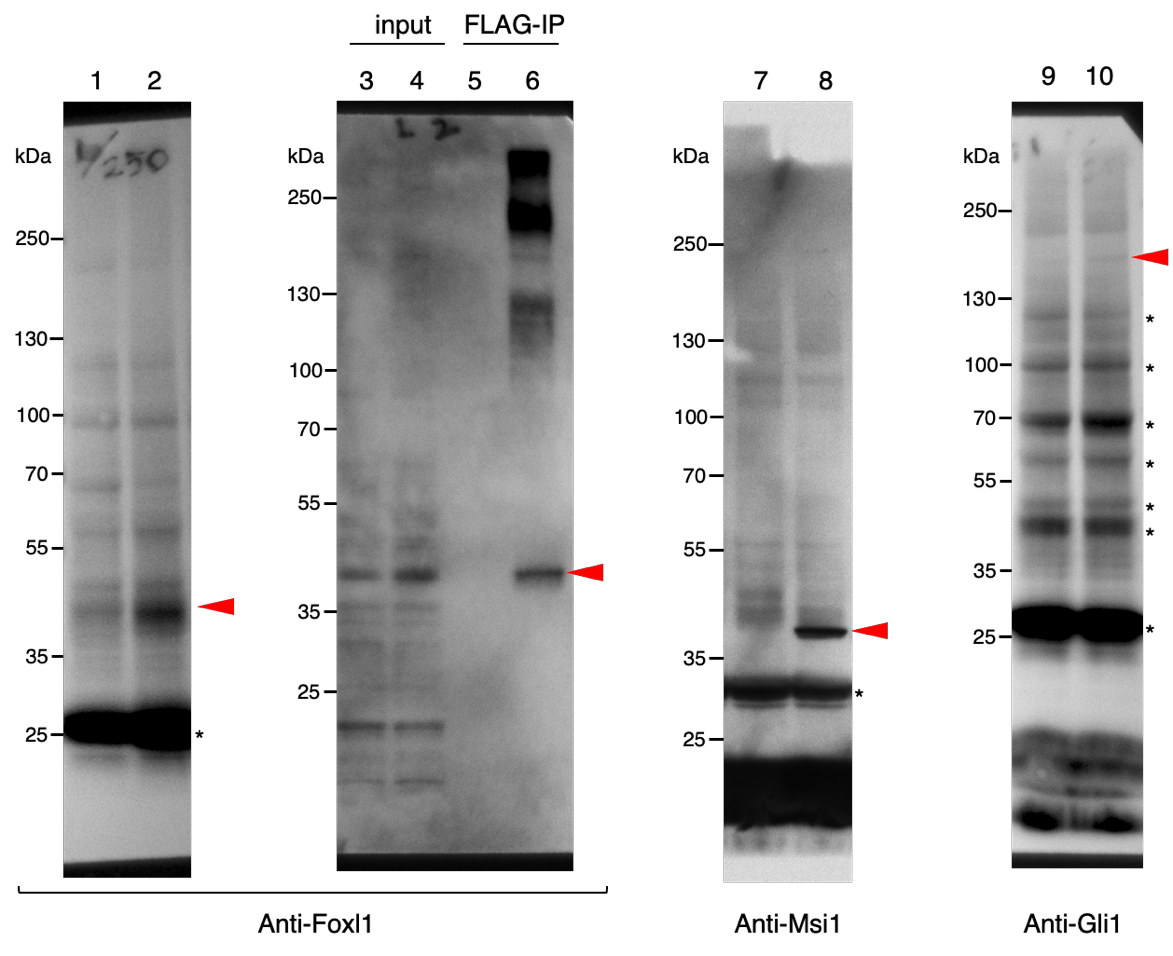


Figure S1. The antibodies specifically recognize *X. laevis* proteins. In vitro-translated (IVT) proteins (1. non-template control, 2. Foxl1.L, 7. non-template control, 8. Msi1.L, 9. non-template control, 10. Gli1.S) produced by TNT T7 or SP6, and input and FLAG-IP samples prepared from *X. laevis* embryos (3, 5. uninjected, 4, 6. FLAG-Foxl1.L mRNA-injected) were analyzed by Western blotting using the indicated antibodies. Arrowheads indicate the specific signals with the expected molecular weights. Asterisks indicate non-specific signals as they are also detected in the negative controls. Note, anti-Foxl1 antibody also detected the endogenous Foxl1 (lane 3). Only a weak signal for Gli1 was detected due to low protein production. Full-length blots are presented.
